# Supplementary material for: Predictors of Free Sugars Intake Trajectories across Early Childhood—Results from the SMILE Birth Cohort Study
Source: Int J Environ Res Public Health. 2024 Feb 2;21(2):174. doi: 10.3390/ijerph21020174 (PMC10888386; doi:10.3390/ijerph21020174)
Supplement: Supplementary file 1 [file ijerph-21-00174-s001.zip › ijerph-2804588-supplementary.pdf]

Supplementary Table S1. Maternal and child characteristics associated with trajectory of free sugars intake generated from PAIRWISE analysis—Unadjusted risk.

	Trajectory 2 Compared with Trajectory 1			Trajectory 3 Compared with Trajectory 1		
	**RRR**	**95% CI**	***p* Value**	**RRR**	**95% CI**	***p* Value**
**Maternal characteristics**						
**Mother's age (*n* = 1372)**	1.00	0.97–1.03	0.908	**0.92**	**0.88–0.97**	**<0.001**
**Mother's highest education level (*n* = 1386)**						
High school (*n* = 248)	1.26	0.78–2.05	0.343	**2.80**	**1.53–5.13**	**<0.001**
Vocational training (*n* = 345)	1.00	0.68–1.47	0.994	1.04	0.58–1.85	0.904
Tertiary education (*n* = 793)	REF			REF		
**Mother's country of birth (*n* = 1367)**						
Australia/ New Zealand (*n* = 971)	REF			REF		
United Kingdom/Ireland (*n* = 49)	0.76	0.33–1.75	0.520	0.89	0.27–2.90	0.848
India (*n* = 103)	0.90	0.48–1.67	0.731	0.86	0.35–2.11	0.748
China (*n* = 52)	0.64	0.30–1.36	0.243	0.42	0.11–1.58	0.198
Asia other (*n* = 105)	1.09	0.56–2.10	0.806	1.25	0.52–3.01	0.623
Others (*n* = 87)	0.83	0.44–1.58	0.566	0.62	0.23–1.73	0.364
**IRSAD decile (*n* = 1369)**	**0.92**	**0.87–0.98**	**0.007**	**0.82**	**0.75–0.89**	**<0.001**
**Household composition (*n* = 1295)**						
Single-parent household (*n* = 74)	1.78	0.70–4.52	0.223	**3.49**	**1.19–10.19**	**0.023**
Two-parent household (*n* = 1221)	REF			REF		
**Mother's pre-pregnancy BMI (kg/m^2) (*n* = 1285)**						
< 25 (*n* = 735)	REF			REF		
25–29.99 (*n* = 298)	1.07	0.70–1.62	0.756	1.14	0.62–2.08	0.672
≥ 30 (*n* = 252)	0.99	0.64–1.53	0.955	1.34	0.73–2.46	0.353
**Child characteristics**						

**Child sex (*n* = 1386)**						
Male (*n* = 744)	REF			REF		
Female (*n* = 642)	0.86	0.62–1.20	0.383	**0.61**	**0.38–0.97**	**0.039**
**Child birth weight (g) (*n* = 1364)**						
<2500 (*n* = 83)	0.86	0.44–1.68	0.664	1.39	0.58–3.32	0.464
2500 -4000 (*n* = 1141)	REF			REF		
> 4000 (*n* = 140)	1.40	0.77–2.55	0.272	1.07	0.45–2.53	0.884
**Number of siblings (1338)**						
None (*n* = 626)	REF			REF		
One (*n* = 497)	1.20	0.83–1.73	0.326	0.90	0.53–1.56	0.715
Two or more (*n* = 215)	1.38	0.82–2.33	0.221	**2.03**	**1.04–3.95**	**0.038**
**Duration of Breastfeeding (weeks)**						
< 17 (*n* = 375)	**1.70**	**1.11–2.61**	**0.016**	**2.97**	**1.62–5.44**	**<0.001**
17–25 (*n* = 117)	1.53	0.81–2.87	0.191	1.61	0.62–4.15	0.326
26–51 (*n* = 275)	**1.77**	**1.06–2.94**	**0.029**	2.01	0.95–4.22	0.067
≥ 52 (*n* = 448)	REF			REF		
**Age of introduction of complementary foods (weeks)**						
< 17 (*n* = 338)	1.65	0.87–3.13	0.126	**2.90**	**1.14–7.34**	**0.025**
17–25 (*n* = 843)	1.16	0.67–2.02	0.603	1.00	0.42–2.37	0.993
≥ 26 (*n* = 116)	REF			REF		

Trajectory 1 Low and increasing; Trajectory 2 Moderate and increasing; Trajectory 3 High and increasing; IRSAD Index of Relative Socio-Economic Advantage and Disadvantage where decile 1 = most socially disadvantaged and decile 10 = most socially advantaged.

Supplementary Table S2. Sensitivity analysis: Maternal and child characteristics associated with trajectory of free sugars intake generated from LISTWISE analysis—Unadjusted risk.

	**Trajectory 2 Compared with Trajectory 1**			**Trajectory 3 Compared with Trajectory 1**		
	**aRRR**	**95% CI**	***p* Value**	**aRRR**	**95% CI**	***p* Value**
**Maternal characteristics**						
**Mother's age** (*n* = 526)	1.01	0.96–1.07	0.608	**0.90**	**0.84–0.97**	**0.007**
**Mother's highest education level (*n* = 527)**						
High school (*n* = 70)	0.95	0.43–2.07	0.890	1.99	0.76–5.21	0.163
Vocational training (*n* = 133)	0.77	0.44–1.36	0.373	0.94	0.42–2.08	0.874
Tertiary education (*n* = 324)	REF			REF		
**Mother's country of birth (*n* = 524)**						
Australia/ New Zealand (*n* = 390)	REF			REF		
United Kingdom/Ireland (*n* = 24)	0.70	0.23–2.17	0.538	0.84	0.18–3.94	0.820
India (*n* = 33)	0.54	0.22–1.33	0.182	0.48	0.12–1.96	0.305
China (*n* = 15)	**0.30**	**0.10–0.92**	**0.036**	0.22	0.03–1.98	0.178
Asia other (*n* = 31)	0.91	0.30–2.75	0.863	1.39	0.35–5.51	0.638
Others (*n* = 31)	0.58	0.22–1.50	0.260	0.74	0.20–2.80	0.660
**IRSAD decile (*n* = 524)**	**0.90**	**0.82–0.99**	**0.024**	**0.81**	**0.72–0.92**	**0.001**
**Household composition (*n* = 525)**						
Single-parent household (*n* = 27)	1.21	0.35–4.19	0.765	2.15	0.49–9.40	0.308
Two-parent household (*n* = 498)	REF			REF		
**Mother's pre-pregnancy BMI (kg/m²) (*n* = 502)**						
< 25 (*n* = 296)	REF			REF		
25–29.99 (*n* = 111)	1.20	0.64–2.27	0.565	0.99	0.39–2.48	0.982
≥ 30 (*n* = 95)	1.12	0.57–2.18	0.751	1.48	0.61–3.63	0.387
**Child characteristics**						

**Child sex (*n* = 527)**						
Male (*n* = 293)	REF			REF		
Female (*n* = 234)	0.85	0.52–1.40	0.532	**0.48**	**0.24–0.97**	**0.040**
**Child birth weight (g) (*n* = 519)**						
<2500 (*n* = 28)	75	0.18–3.17	0.699	0.78	0.26–2.39	0.667
2500 -4000 (*n* = 427)	REF			REF		
> 4000 (*n* = 64)	0.75	0.23–2.48	0.642	1.36	0.55–3.33	0.505
**Number of siblings (511)**						
None (*n* = 253)	REF			REF		
One (*n* = 188)	1.36	0.81–2.31	0.247	0.63	0.28–1.43	0.271
Two or more (*n* = 70)	**4.37**	**1.30–14.63**	**0.017**	**6.62**	**1.75–25.02**	**0.005**
**Duration of Breastfeeding (weeks)**						
< 17 weeks (*n* = 116	1.32	0.68–2.54	0.413	1.82	0.75–4.44	0.182
17–25 weeks (*n* = 55)	1.11	0.50–2.48	0.800	0.57	0.14–2.35	0.438
26–51 weeks (*n* = 116)	**2.14**	**1.01–4.51**	**0.046**	2.40	0.91–6.36	0.078
≥ 52 weeks (*n* = 212)	REF			REF		
**Age of introduction of complementary foods (weeks)**						
< 17 weeks (*n* = 126)	1.78	0.71–4.49	0.221	2.64	0.74–9.45	0.136
17–25 weeks (*n* = 354)	1.63	0.73–3.63	0.236	1.19	0.37–3.86	0.774
≥ 26 weeks (*n* = 44)	REF			REF		

Trajectory 1 Low and increasing; Trajectory 2 Moderate and increasing; Trajectory 3 High and increasing; IRSAD Index of Relative Socio-Economic Advantage and Disadvantage where decile 1 = most socially disadvantaged and decile 10= most socially advantaged.

Supplementary Table S3. Sensitivity analysis: Maternal and child characteristics associated with trajectory of free sugars intake generated from LISTWISE analysis—Adjusted risk (*n* = 483).

	**Trajectory 2 Compared with Trajectory 1**			**Trajectory 3 Compared with Trajectory 1**		
	**aRRR**	**95% CI**	***p* Value**	**aRRR**	**95% CI**	***p* Value**
**Maternal characteristics**						
**Mother's age**	1.01	0.96–1.07	0.672	**0.90**	**0.82–0.98**	**0.011**
**IRSAD quintile**	**0.89**	**0.80–0.98**	**0.018**	**0.83**	**0.72–0.96**	**0.009**
**Child characteristics**						
**Child sex**						
Male (*n* = 263)	REF			REF		
Female (*n* = 220)	0.86	0.51–1.46	0.578	**0.45**	**0.21–0.99**	**0.047**
**Number of siblings**						
None (*n* = 241)	REF			REF		
One (*n* = 175)	1.48	0.83–2.64	0.181	1.04	0.42–2.53	0.939
Two or more (*n* = 67)	**4.41**	**1.28–15.23**	**0.019**	**10.65**	**2.55–44.38**	**0.001**
**Duration of Breastfeeding (weeks)**						
< 17 weeks (*n* = 113)	1.35	0.69–2.66	0.386	1.52	0.60–3.88	0.378
17–25 weeks (*n* = 53)	1.11	0.48–2.54	0.814	0.27	0.05–1.43	0.124
26–51 weeks (*n* = 112)	2.15	1.00–4.61	0.050	2.18	0.79–6.06	0.134
≥ 52 weeks (*n* = 205)	REF			REF		

Likelihood Ratio Test Chi-Square = 52.767, df = 16, $p < 0.001$; Trajectory 1 Low and increasing (*n* = 69, 14.3%); Trajectory 2 Moderate and increasing (*n* = 362, 74.9%); Trajectory 3 High and increasing (*n* = 52, 10.8%); IRSAD Index of Relative Socio-Economic Advantage and Disadvantage where decile 1 = most socially disadvantaged and decile 10 = most socially advantaged.
